# Dermatofibrosarcoma protuberans, a rare but locally aggressive tumor on finger: clinical and aeromedical considerations

**DOI:** 10.3109/23320885.2014.995185

**Published:** 2015-01-06

**Authors:** Kwo-Tsao Chiang, Shih-Yu Lee, Hsin Chu

**Affiliations:** ^a^ Aviation Physiology Research Laboratory, Gangshan Branch of Armed Forces Kaohsiung General Hospital, Kaohsiung, Taiwan, Republic of China; ^b^ Institute of Aerospace and Undersea Medicine, School of Medicine, National Defense Medical Center, Taipei, Taiwan, Republic of China

**Keywords:** Dermatofibrosarcoma protuberans, Mohs surgery, waiver, aeromedical disposition

## Abstract

Dermatofibrosarcoma protuberans (DFSP) is a rare, slow growing, locally infiltrative tumor of intermediate malignancy. It is mostly found on the trunk and head, rarely on hands. The course of evaluation and treatment of a young pilot with DFSP on left middle finger is reported. The clinical issues and aeromedical considerations of this rare tumor is discussed.

## Introduction

Cancer is considered a disqualifying condition for civilian pilots. Resumption of flying status for pilots diagnosed with cancer can only be considered after the successful removal of the tumor, the completion of all forms of treatment, absence of metastatic disease and complications as well as maintenance of functions, without jeopardizing flight safety. Dermatofibrosarcoma protuberans (DFSP) is an extremely rare, slow growing and locally aggressive dermal neoplasm of mesenchymal origin [1]. Most DFSP occurs on the trunk, proximal extremities, and head and neck regions [2]. Only several cases have been reported to occur on fingers. Hand function is essential to successfully control the airplane. DFSP is characterized by local aggressiveness and if not completely removed, it easily recurs [3]. We report on a young pilot with a DFSP over the left middle finger. The course of evaluation and treatment involving three surgeons and two hospitals highlights the importance of early planning and staged surgery in the treatment of this rare tumor.

## Case report

**Figure 1.**
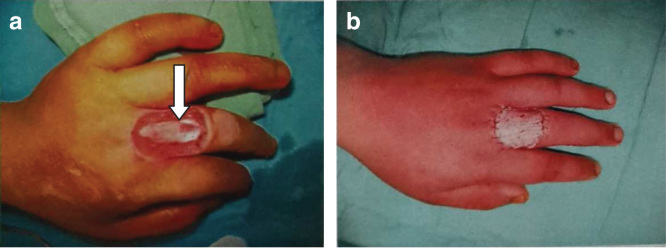
**Temporary management of the wound. (*a*) Exposed extensor tendon over dorsal aspect of proximal phalanx of left middle finger. (*b*) Transient coverage of wound with cadaveric skin graft. The white arrow indicated exposed tendon.**

**Figure 2.**
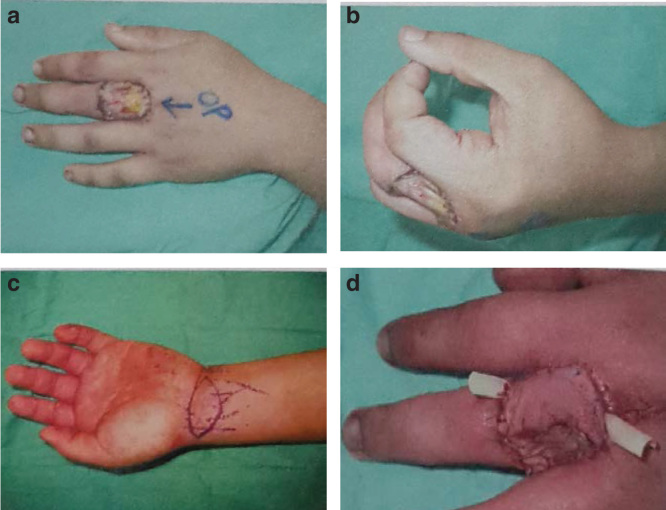
**Finger reconstruction by using the SPBRA flap. (*a*) Preoperative defect of the left middle finger (antero-posterior view). (*b*) Preoperative defect of the left middle finger (lateral view). (*c*) Flap design. (*d*) Postoperative view.**

A healthy, 29-year-old male commercial airline pilot without individual or family history of skin cancer or other malignancies noticed a symptom-free movable nodular mass over the dorsal proximal phalanx of his left middle finger, near the metacarpophalangeal joint. The tumor gradually enlarged to 2 cm in diameter in two years and was associated with local erythematous change. An excision biopsy was performed by a general surgeon in a medical center. Histopathological examination revealed a nodular neoplastic lesion composed of spindle-shaped cells with frequent mitotic figures. Immunohistochemical stain was negative for actin but positive for CD34. Thin collagen fibers crisscrossing between the spindle cells stained positive with Masson trichrome. The pathological diagnosis was consistent with DFSP. Due to the close proximity of surgical margins to the tumor mass and the characterized highly recurrent nature locally of the tumor, a wider excision was strongly suggested by the general surgeon. The pilot visited the plastic surgery outpatient clinics in another hospital 3 weeks later for a second opinion. A wide excision and split-thickness skin graft with donor from left palm hypothenar region were done. Width of the surgical margin was not mentioned in the medical record. A pathology report revealed residual DFSP in the lower dermis and subcutis region of the skin surrounding the previous wound. Two weeks after his second surgery, he was admitted to the previously-mentioned medical center where a plastic surgeon carried out a wide excision of the skin and soft tissue around the previous wound. About 1 cm margin around the lesion was excised. The depth of the defect was 0.5 cm, subcutaneous tissue was involved, but tendons were spared. In order to make sure the surgical margins are free from tumor invasion, even with the frozen section, the reconstruction was delayed until definitive pathology report. The defect over the left middle finger was transiently covered with a cadaveric skin graft after confirming free of tumor invasion over surgical margins and base with intra-operative frozen section analysis (Figure 1). Functional reconstruction was achieved 1 week later by tissue coverage, using a superficial palmar branch of the radial artery (SPBRA) free flap [4]. The SPBRA bifurcates from the main trunk of radial artery about 2 cm proximal to the distal wrist crease. Usually, one or two concomitant veins accompanied the SPBRA. After marking the SPBRA, an elliptical shaped 35 × 25 mm flap over the volar surface of distal forearm was designed to facilitate donor site closure (Figure 2). A proximal incision was made to explore the SPBRA after confirmation by Doppler flowmetry. The flap was elevated, the SPBRA and its two concomitant veins were anastomosed to the digital artery and digital dorsal veins, respectively, under microscope. The flap was harvested as a sensate flap by incorporating palmar cutaneous branch of the median nerve into the flap. The donor site was closed without complication or obvious scarring. The recipient site achieved a full range of motion and showed a good contour. Both dynamic and static two-point spatial discriminations were tested and adequate protective sensation was attained.

The pilot did not disclose the whole process of tumor evaluation and management until his next annual aircrew physical examination. Upon doing so, he was grounded immediately. After reviewing pertinent medical records and being closely followed-up for 9 months without recurrence since the last operation, a waiver was granted and the pilot returned to flight status. To date, there has been no evidence of tumor recurrence 4 years after a complete surgical resection.

## Discussion

For commercial airline pilot to successfully control of the airplane, there should be no major impairment of the three basic types of functions of the hand: to grasp cylindrical objects; to pinch by tip, pulp or by lateral pressure; to hook. Complete intact sensibility and good finger and thumb movements on both sides are also essential for operation of computer display and keyboards.

DFSP is an extremely rare soft tissue sarcoma mostly diagnosed in young and middle-aged adults, with estimated incidence ranging from 0.8 to 5 per million [1]. Analysis of case series showed that DFSP is mainly discovered over the trunk (50%), proximal extremities (20–35%) as well as the head and neck region (10–15%). It is rarely seen on distal extremities and only a few cases of DFSP on fingers or hands had been reported in the English language literature published in peer-reviewed journals (Table 1). The rate of metastasis is estimated to be between 0.5% and 5% [1]. Because of a propensity for local recurrence and the ability to rarely metastasize, DFSP is rated as intermediate malignant according to recent WHO classifications [5].

**Table 1.** **Characteristics of DFSP in hands or fingers reported in the literature.**

References	Age & sex	Anatomic site & size (cm)	Treatment	Follow up and status
Taylor & Helwig, 1962 [12]	NA	Three cases: forearm, wrist, dorsal hand; (NA)	Wide local resection	NA
Chiang *et al.*, 1993 [13]	51 F	Hypothenar region of hand (1.5)	Local excision	First recurrence 10 yrs after excision, second recurrence 3 yrs later.
Reimann *et al*., 2007 [14]	54 F	Left second finger (1.5)	Wide excision	2 recurrences/2 yrs; ND at 9 yrs
Chiang, Lee & Chu, 2014	29 M	Left middle finger (2)	Excision & reconstruction	ND at 4 yrs

Abbreviations: DFSP = Dermatofibrosarcoma protuberans; F = Female; M = Male; NA = Not available; ND = No evidence of disease; yrs = Years.

DFSP should be suspected in patients with histories of firm, slow-growing cutaneous nodules over the trunk, proximal limbs, and head and neck region. An accurate diagnosis of DFSP can be achieved by means of an open biopsy combined with histological analysis and immunohistochemical staining. Histologically, DFSP is composed of relatively uniform, spindle-shaped tumor cells with prominent nuclei and high mitotic index, grouped together and forming short fascicles with a storiform pattern. Tumor cells can be identified and differentiated by immunoreactivity to CD34 (a broad-spectrum endothelium-associated marker), hyaluronate, vimentin, and the absence of immunoreactivity to factor XIIIa and S100 [6]. DFSP is characterized by neoplastic finger-like villous projections infiltrating into the subcutaneous tissues up to 3 cm peripherally, intermingled with normal tissues. Thus, the histological tumor-free margins may differ greatly from gross margins and a biopsy can easily result in incomplete evaluation and non-radical excision of the tumors, as occurred in this case. To decide the safety margin for resection represents a great challenge, as failure of complete excision leads to local recurrence [3]. Furthermore, since metastases seldom develop in the absence of antecedent local recurrence, local recurrence is also considered a risk for distant metastasis [7].

The extent and completeness of the resection decide the recurrence rate and may even affect the metastatic rate in DFSP. A1.6-cm histological margin was considered adequate for complete local control and that most patients can be operated on in one stage. Pooled data from 1443 DFSP patients who underwent local excision revealed that with wide surgical margins of 2–3 cm, average tumor recurrence rate was 7.3%, compared with 20% recurrence using regular excision [1]. Mohs micrographic surgery has recently been applied to treat DFSP to preserve vital tissues and facilitate reconstruction. This technique examines the complete peripheral and deep margins of the tumor utilizing mapped horizontal frozen sections of excised tissue, allows microscopic assurance of tumor-free margins and complete excision of tumors [8]. Because of the ability to target positive margins, the recurrence rate of DFSP after Mohs micrographic surgery was reduced to 1%, according to pooled data of 444 patients [1]. DFSP is best treated in specialist centers by experienced surgeons and pathologists. If DFSP develops on extremities or the acral region, early involvement of plastic surgeons is mandatory [9]. Multivariate analysis of 122 DFSP cases in a cancer hospital revealed that acral location, along with fibrosarcomatous change, are the main factors that are significantly associated with shorter recurrence-free survival. Aggressive treatment is recommended for those cases [10].

Cancer is considered a disqualifying condition according to the policy of the Civil Aviation Medical Center, Civil Aeronautics Administration of the Republic of China, as it is in most aeromedical regulations of the world. Pilots diagnosed with cancer are obligated to report it to Civil Aviation Medical Center. They will be grounded and their medical certificate suspended. Re-issuance of an airman’s medical certificate is not considered until after documentation of successful removal of the tumor and completion of treatment. All cases will be sent to the Aviation Medicine Advisory Committee for evaluation after follow-up time of at least 1 year to ensure absence of metastasis. Certain cancers with low metastatic potential that have been completely excised may be evaluated earlier. In the current case of DFSP, the pilot returned to flight status 9 months after surgery.

The SPBRA flap was chosen on this pilot to provide a thin, glabrous and pliable skin flap for covering medium to large finger defects with exposed tendon. Free flaps including anterolateral thigh flap and groin flap requires prolonged operative time and revision surgery for debulking. Pedicle flaps may not be sufficient to cover large soft tissue loss, and mostly provide only skin coverage. Procedures such as nerve or tendon grafting might be needed for fully functional reconstruction. There are several obvious advantages of the SPBRA flap: harvesting the flap is quick and easy; the flap provides thin, pliable and hairless skin; involving simple brachial block anesthesia; no major vessel is sacrificed; only one operative field is required; and allowing sensory recovery. According to a recent study, SPBRA flap is comparable to other techniques (i.e., arterialized venous flap, posterior interosseous perforator flap, ulnar artery perforator free flap) using flap from ipsilateral extremity for finger reconstruction [11].

For complete treatment of the DFSP on finger, the pilot had four operations in 2 months by three doctors from two hospitals. If he had informed the aeromedical specialist of the Civil Aviation Medical Center of the pathologic report after his initial excision biopsy, timely medical advice or opinions could have been provided. A multidisciplinary care team from a single institution can be organized to provide proper diagnosis and treatment of the illness while taking into consideration the subject’s pilot career, as well as flight safety. For DFSP, the extent of the resection should not be compromised by the reconstructive options available. Fortunately, the final operation on this pilot utilizing mapped horizontal frozen sections of excised tissue assured tumor-free margins. After successful reconstructive procedure, the pilot’s finger was preserved and his left hand was able to function normally without neurologic deficits.

## Conclusion

In summary, this is an extremely unusual case of a soft tissue sarcoma arising in an unusual place, which, unfortunately, was inadequately excised and subsequently required three surgeries by different surgeons in different institutions. DFSP on a finger is associated with shorter recurrence-free survival. Current treatment involves surgery and radiotherapy. The clinical scenario in this particular case emphasizes the need for adequate initial evaluation by a multidisciplinary team of reconstructive surgeons and pathologists familiar with this sarcoma. With meticulous preoperative planning and postoperative analysis of histological tumor-free margins in millimeters in all directions, the risk of local recurrence and tumor progression can be minimized. DFSP is best treated in specialist centers. Early referral is highly recommended in order to achieve good oncologic outcomes.
